# Alka Dwivedi: developing a cancer therapy for all

**DOI:** 10.2471/BLT.24.030924

**Published:** 2024-09-01

## Abstract

Alka Dwivedi talks to Gary Humphreys about developing a new form of CAR T-cell therapy in collaboration with clinicians with a view to improving treatment outcomes and lowering costs.

*Q* ***:*** *How did you come to your focus on immunotherapy?*

A**: **Well, I was always interested in therapy, in healing, and even considered becoming a doctor for a while, but I suppose that the scientist in me won out. I pursued a master’s degree in biotechnology at Nagpur University in Maharashtra, where I explored the application of bioactive plant extracts in cancer treatment, a fairly new field at that time. I then went on to work at a biotech company where I received industrial training on the application of molecular tools in the field of new drug discovery, and became very interested in their application to potential drugs for the treatment of cancer.

*Q: What sparked your particular interest in CAR T-cell-based therapies?*

A: As a young biotechnician it was always on my radar, but I would say that my passion for CAR T-cell therapies was really ignited at the Indian Institute of Technology (IIT) in Mumbai, where I had the good fortune to work with Professor Rahul Purwar, a leading light and pioneer in cancer immunotherapy and skin immunology research. He took a keen interest in CAR T-cell therapy, which was really starting to gain momentum at that time.

*Q: Can you explain what CAR T-cell therapy is in simple terms?*

A: It’s a form of therapy that harnesses the body’s T-cells, a type of white blood cell that plays a crucial role in the immune system, particularly in the adaptive immune response. Putting it in the simplest terms, T-cells have evolved special receptors that bind to different antigens and – depending on the type of T-cell in question – this binding can result in the production of cytokines, small proteins that play a role in cell signalling. CAR T-cell therapy involves extracting T-cells from a patient’s blood, genetically modifying them to make a new kind of antigen receptor – a chimeric antigen receptor or CAR – that targets specific cancer cell proteins. These modified T-cells are then reintroduced into the patient’s bloodstream where they target and kill the cancer cells. The approach was first conceptualized and developed in the late 1980s in Israel, and since then thousands of scientists around the world have worked in the area, including Carl June’s research group in the USA, which conducted the first CAR T-cell therapy clinical trial in 2011. In 2015, when I went to the IIT, some new CAR T-cell therapies were going through clinical trials in the USA, and Professor Purwar and I, along with Dr Gaurav Narula from Tata Memorial Hospital in Mumbai, started talking about finding ways to improve the therapy for use in India.

*Q: What needed to be improved?*

A: The therapies going through clinical trials in the USA looked really promising as treatments for leukaemia and lymphoma, but they were known to have significant side-effects including something called cytokine release syndrome, the massive release of cytokines into the bloodstream that can result in symptoms ranging from a mild headache and fever to difficulty breathing and life-threatening multi-organ dysfunction. Another issue was their cost. At that time, it was assumed that they would cost hundreds of thousands of dollars if they finally got approval (they ended up costing between 373 000 United States dollars (US$) and US$ 475 000 per treatment) which meant that they were unlikely to benefit most cancer patients in India. This was of major concern to us and not just at an abstract, academic level. Cancer was becoming a significant public health issue in India, with new diagnoses standing at around 980 000 in 2010 and trending upwards, but being so close to the Tata Memorial Hospital, one of the country’s leading cancer centres, we all saw patients with cancer every day. I remember the first time I went to the hospital and saw the lines of patients, many of them children, suffering with leukaemia or lymphoma. That had a profound effect on me.

“[We achieved] a 90% reduction in cost relative to treatments currently available.”

*Q: How did you go about improving CAR T-cell therapies?*

A: We started out by working on ways to optimize the process of CAR T-cell production. Specifically, I was focusing on the production of the viral vector we needed to carry and insert specific genes into the patients’ T-cells. We used a lentivirus for the purpose – a type of retrovirus that can insert itself into the DNA of host cells which makes it a powerful gene therapy tool once the lentivirus is rendered harmless. The technology has been around for some time, but it still presents considerable challenges to the would-be bioengineer, partly because producing such vectors is complex, and partly because it is necessary to grow a significant volume of viral particles to be able to successfully modify the T-cells. At IIT, this was particularly challenging because our lab was new and, in many instances, we were setting up the relevant equipment and processes for the first time. So, the learning curve was very steep, and the first few years were really challenging. Very often I would improve one part of the process only to have issues arise elsewhere. Then in 2017, I was able to spend some time at the National Cancer Institute in USA where I got some very helpful technical advice from experts, and by the end of the following year I had made significant progress in viral vector production and CAR T-cell generation. We got to the point where we were able to start testing our CAR T-cells on mice, achieving high rates of remission, and received regulatory approval to advance to clinical trials. We were then faced with the challenge of scaling up production and optimizing all the processes in preparation for the phase I trial. This meant building a facility at IIT Bombay, which we did based on the good manufacturing practices established by the World Health Organization to ensure sterility and quality of the production, and by June 2021 we were ready. We started phase I clinical trials with the informed consent of patients from the Tata Memorial Hospital who had advanced lymphoma or leukaemia.

*Q: What were the headline results of the trials?*

A: The overall response rate of our phase I/II clinical trial was 70% (23 out of 33), with responsive patients showing a significant reduction in cancer presence and approximately half achieving complete remission. We created a therapy that is not only effective but also results in the production of fewer cytokines, lowering the incidence of cytokine release syndrome and neurotoxicity. This improves outcomes and patients’ experience of the treatment, and also reduces the overall treatment cost. Additionally, we were able to bring down production costs by making the lentivirus and the CAR T-cells in India, using a vertical, integrated business model. The result was a treatment costing around US$ 50 000, a 90% reduction in cost relative to treatments currently available in the US. In October 2023, the Central Drugs Standard Control Organization, which is India's equivalent of the US Food and Drug Administration (FDA), granted approval to NexCAR19 (the product’s name), making it India's first authorized CAR-T cell therapy.

“We intend to expand beyond India, targeting low- and middle-income countries.”

*Q: Average annual income in India is around US$ 2000, with many earning less than that. Is US$ 50 000 affordable for most people?*

A: Clearly affordability remains a challenge, but we hope to bring the cost down further as we ramp up NexCAR19 production and the number of treatments we can do. To date, we have completed over 150 NexCAR19 patient infusions and we expect to treat 400 to 500 patients next year. A larger production facility is being constructed on the outskirts of Mumbai, with the aim of increasing treatment capacity to about 1200 patients per year. The new facility will include improved facilities for producing viral vectors and CAR T-cells, alongside a larger quality control area to expedite the readiness of products for patient use. While scale-up should enable us to bring down the cost further, the insurance sector and government clearly have an important role to play in bringing down out-of-pocket expenses for individual patients.

*Q: Do you have plans to make the therapy available internationally?*

A: We intend to expand beyond India, targeting low- and middle-income countries where treatments are still unavailable and unaffordable. ImmunoACT, the biotech company that we created to commercialize the product, has signed a memorandum of understanding with the Government of Mexico, and we are exploring collaborations in other regions.

*Q: You are currently working as a postdoctoral Research Fellow at the National Cancer Institute in Bethesda, Maryland. What brought you there?*

A: After earning my PhD in 2021, I was keen to explore the CAR-T field further, working with the experts who were so helpful to me in 2017, and that is what I am doing, essentially continuing my research and training. 

*Q: Do you have any plans to return to India?*

A: That is my intention. I am very much enjoying my time here and have to say I really appreciate how efficient and smooth everything is. For example, here I just take the elevator down to see the mice I am working with, whereas in Mumbai I had to drive for an hour in Mumbai traffic! But, yes, my plan is to return to India and continue working in this field. Currently NexCAR19 is available for patients with blood cancers only. Other applications of the therapy are now being explored, including treatments for breast, lung and pancreatic cancers. I couldn’t be more excited about the prospect of contributing to that exploration.

